# Neuropsychological effects of shunting in iNPH – determining major and minor responses

**DOI:** 10.1186/2045-8118-12-S1-O53

**Published:** 2015-09-18

**Authors:** Maria Wallin, Mats Tullberg, Carsten Wikkelsö, Per Hellström

**Affiliations:** 1 Institute of neuroscience and physiology, Sweden

## Introduction

iNPH is characterized by a wide range of neuropsychological deficits. The severity of impairment is associated with that of gait, balance and continence disturbances, and correlations between results on disparate neuropsychological tests are far stronger than among healthy; a dedifferentiation of functions.

Following surgery, performance at group level, is significantly improved and remains stable for at least the first year of treatment. Importantly, however, shunted iNPH patients still perform well below normal levels.

The magnitude of change varies substantially, some patients showing only modest advances or even further deterioration, whereas others show considerable improvements on the majority or all administered tests.

In this study we compared patients belonging to these two extremes, aiming to predict neuropsychological benefits of surgery.

## Methods

Consecutive iNPH patients (N=235) underwent pre- and postoperative assessments of learning (Rey Auditory Verbal Learning test; RAVLT), dexterity (Grooved pegboard), basic processing speed (Stroop, color naming task) and executive control (Stroop, interference task). For each test the patients were divided into four groups (Q1-Q4) according to the quartile limits of the distributions of Reliable Change Indices (RCIs). Q1 and Q4, i.e. the least and the most improved, were compared with regard to preoperative characteristics pertaining to demography, other iNPH manifestations and other findings in neurological and physiotherapeutic examinations, and CSF dynamics. For each set of analyses they were also compared with regard to the changes (RCI scores) and postoperative performances on the remaining three tests.

## Results

Patients who showed substantial positive changes in one neuropsychological function did so in the others as well, and vice versa. For learning and dexterity, neither demographic, nor clinical or CSF dynamics predicted changes following treatment. In the group who showed the greatest improvement of basic processing speed, preoperative gait disorders were more severe, and paratonia was more frequent than among the least improved. Those with greatest positive changes in executive control also had more severe gait disorders and more frequent paratonia, and, in addition, more marked problems with turning, balance and continence, and a lower general intellectual level.

## Conclusion

Neuropsychological improvement following shunt treatment is consistent across functions. Statistically reliable and meaningful changes in learning and dexterity can not be predicted from demography, severity in other iNPH symptoms or CSF dynamics. Changes of basic processing speed and executive control, however, do appear possible to foresee on the basis of preoperative findings.

